# Hyperpolarized ^13^C magnetic resonance imaging for noninvasive assessment of tissue inflammation

**DOI:** 10.1002/nbm.4460

**Published:** 2020-12-08

**Authors:** Stephanie Anderson, James T. Grist, Andrew Lewis, Damian J. Tyler

**Affiliations:** ^1^ Department of Physiology, Anatomy and Genetics University of Oxford Oxford UK; ^2^ Division of Cardiovascular Medicine, Radcliffe Department of Medicine University of Oxford Oxford UK; ^3^ Department of Radiology, The Churchill Hospital Oxford University Hospitals Trust Headington UK

**Keywords:** hyperpolarized magnetic resonance imaging, inflammation

## Abstract

Inflammation is a central mechanism underlying numerous diseases and incorporates multiple known and potential future therapeutic targets. However, progress in developing novel immunomodulatory therapies has been slowed by a need for improvement in noninvasive biomarkers to accurately monitor the initiation, development and resolution of immune responses as well as their response to therapies. Hyperpolarized magnetic resonance imaging (MRI) is an emerging molecular imaging technique with the potential to assess immune cell responses by exploiting characteristic metabolic reprogramming in activated immune cells to support their function. Using specific metabolic tracers, hyperpolarized MRI can be used to produce detailed images of tissues producing lactate, a key metabolic signature in activated immune cells. This method has the potential to further our understanding of inflammatory processes across different diseases in human subjects as well as in preclinical models. This review discusses the application of hyperpolarized MRI to the imaging of inflammation, as well as the progress made towards the clinical translation of this emerging technique.

## Graphical abstract

Inflammation is a central mechanism underlying numerous diseases and incorporates multiple known and potential future therapeutic targets. Hyperpolarized magnetic resonance imaging (MRI) is an emerging molecular imaging technique with the potential to assess immune cell responses by exploiting characteristic metabolic reprogramming in activated immune cells. This review discusses the application of hyperpolarized MRI to the imaging of inflammation, as well as the progress made towards the clinical translation of this emerging technique.

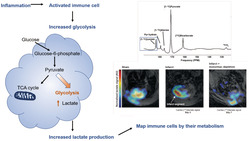

**Abbreviations used**
ATP: adenosine triphosphate
CNS: central nervous system
CoA: coenzyme A
CX3CR1: immune receptor CX3 chemokine receptor 1
DAMPs: damage‐associated molecular patterns
2‐DG: 2‐deoxyglucose
DNP: dynamic nuclear polarization
^18^F[FDG]: ^18^F‐flurodeoxyglucose
FLAIR: FLuid Attenuated Inversion Recovery
LDH: lactate dehydrogenase
LPS: lipopolysaccharide
MBP: myelin basic protein
MCT: monocarboxylate transporter
MDSCs: myeloid‐derived suppressor cells
MI: myocardial infarction
MR: magnetic resonance
MRI: magnetic resonance imaging
mRNA: messenger ribonucleic acid
MS: multiple sclerosis
NADH: nicotinamide adenine dinucleotide
NMO: neuro myelitis optica
PDH: pyruvate dehydrogenase
PDK1: pyruvate dehydrogenase kinase 1
PET: positron emission tomography
PRRs: pattern recognition receptors
pUUO: partial Unilateral Ureteral Obstruction
TBI: traumatic brain injury
TCA: tricarboxylic acid
TSPO: translocator protein

## 1 INTRODUCTION

The primary role of the immune system is to protect the host organism from pathogenic microbial organisms while minimizing self‐injury as a result of effector responses directed against pathogens. Broadly, the immune system utilizes two complementary arms to achieve this goal: innate responses (representing the first line of defense and incorporating physical tissue barriers and specialized proteins and cells that respond to conserved signals indicating tissue injury or infection) and adaptive responses (that permit exquisite selectivity and memory to specific pathogens). Although the immune system has evolved elegant mechanisms to discriminate self from nonself to minimize the risk of self‐injury, dysregulation of immunity leading to adverse inflammation represents a central mechanism of pathogenesis in numerous diseases and provides a host of potential targets for novel therapeutics.

Dysregulated immune responses are important in essentially all body systems, although they have probably been historically underappreciated in cardiovascular and neurologic diseases.^1^, ^2^, ^3^, ^4^ Activation of inflammatory pathways is seen in atherosclerosis,^5^ heart failure,^6^ diabetic cardiomyopathy,^7^ rheumatoid arthritis,^8^ pulmonary inflammation,^9^ renal inflammation,^10^ Crohn's disease^11^ and colitis,^12^ among many others. Neurological diseases such as multiple sclerosis (MS),^13^ Parkinson's^14^ and Alzheimer's disease^15^ are also affected by chronic inflammation. Better ways to assess the initiation, development and resolution of immune responses, using repeated measures with spatial localization, could improve our understanding of the role of the immune system and accelerate the development of new therapies. While peripheral blood assays for serological measurements of immune cell counts or cytokine levels are the current clinical gold standard for monitoring inflammatory response in some diseases, they offer limited insight into spatial localization of inflammatory activity at the specific tissue of interest, and the development of better techniques for inflammation imaging is an important goal.

Existing magnetic resonance imaging (MRI) techniques provide exquisite structural imaging that can be used to assess the consequences of inflammatory disease but not the inflammatory process itself. For example, in the heart, the consequences of dysregulated inflammation (such as myocardial edema, scar formation, fibrosis and impairment of cardiac function) can be routinely assessed using conventional MRI and parametric mapping.^16^, ^17^ In MS, white matter demyelination, as determined by magnetization transfer‐weighted imaging, the rate of white matter lesion formation, as derived from T_2_ FLuid Attenuated Inversion Recovery (FLAIR)‐weighted imaging, and cerebral atrophy, determined from T_1_‐weighted imaging, all reflect late‐stage consequences of inflammatory activity within the central nervous system (CNS).^18^

^18^F‐flurodeoxyglucose (^18^F[FDG])–positron emission tomography (PET) is used to probe inflammation on the principle that activated and increased populations of leukocytes demonstrate increased glucose metabolism and hence increased uptake of ^18^F[FDG].^19^, ^20^, ^21^ This approach has been applied in studies involving human patients and is used as a biomarker of anti‐inflammatory therapies and in assisting diagnosis of inflammation of unknown origin.^22^, ^23^, ^24^ However, with respect to imaging some immune cell populations, ^18^F[FDG] may not be the most reliable imaging agent.^25^ Macrophages, for example, express large quantities of glucose‐6‐phosphatase, which is associated with accelerated efflux of FDG from activated macrophages, limiting its capacity as an uptake biomarker of their activity.^26^ ^18^F[FDG] has well‐recognized limitations for assessing inflammation in organs with high background glucose uptake, such as the heart or brain; for example, ^18^F[FDG] has been compared with the PET agent, gallium‐68‐DOTATATE (^68^Ga‐DOTATATE), for imaging inflammation in atherosclerosis, where it was found to be significantly poorer at predicting high‐risk coronary artery lesions than ^68^Ga‐DOTATATE.^27^ Gd‐based contrast imaging is also able to assess inflammation associated with cardiac, neurological and gastrointestinal inflammation.^28^, ^29^, ^30^ ^11^C‐choline is an imaging agent with useful applications in identifying an unknown origin of inflammation in a clinical diagnostic setting.^31^ Choline is a biosynthetic material required to synthesize the phospholipids of the cell membrane; its uptake is increased in highly proliferative tissues, and its utility as a hyperpolarized agent has been demonstrated for applications assessing brain tumor and prostate cancer.^32^, ^33^

Currently, no routine MR techniques are clinically available to image immune cell activity directly, and one reason for this is the intrinsic limitation in terms of signal‐to‐noise ratio of MRI approaches at biological temperatures and clinically achievable field strengths. Hyperpolarized MR refers to a range of techniques that can provide contrast agents with signal‐to‐noise ratio improvements of up to 4‐5 orders of magnitude over thermal equilibrium. Hyperpolarized MR has now been widely used in preclinical models to study tissue metabolism, demonstrating the ability of the technique to exploit changes in metabolic flux as an indicator of physiological and pathological processes.^34^, ^35^, ^36^, ^37^, ^38^ It is also now apparent that inflammatory responses can be monitored through alterations away from the normal quiescent metabolic activity of the cells of the immune system. Hyperpolarized MRI can be used to examine metabolic changes in immune cells in vivo giving real‐time insights into inflammatory responses. This review explores recent progress in the field of hyperpolarized MRI that highlights its potential for application in examining inflammatory physiology and its response to treatment.

## 2 POTENTIAL TO USE HYPERPOLARIZED MR TO DETECT INFLAMMATION VIA CHANGES IN IMMUNE CELL METABOLISM

**FIGURE 1:**
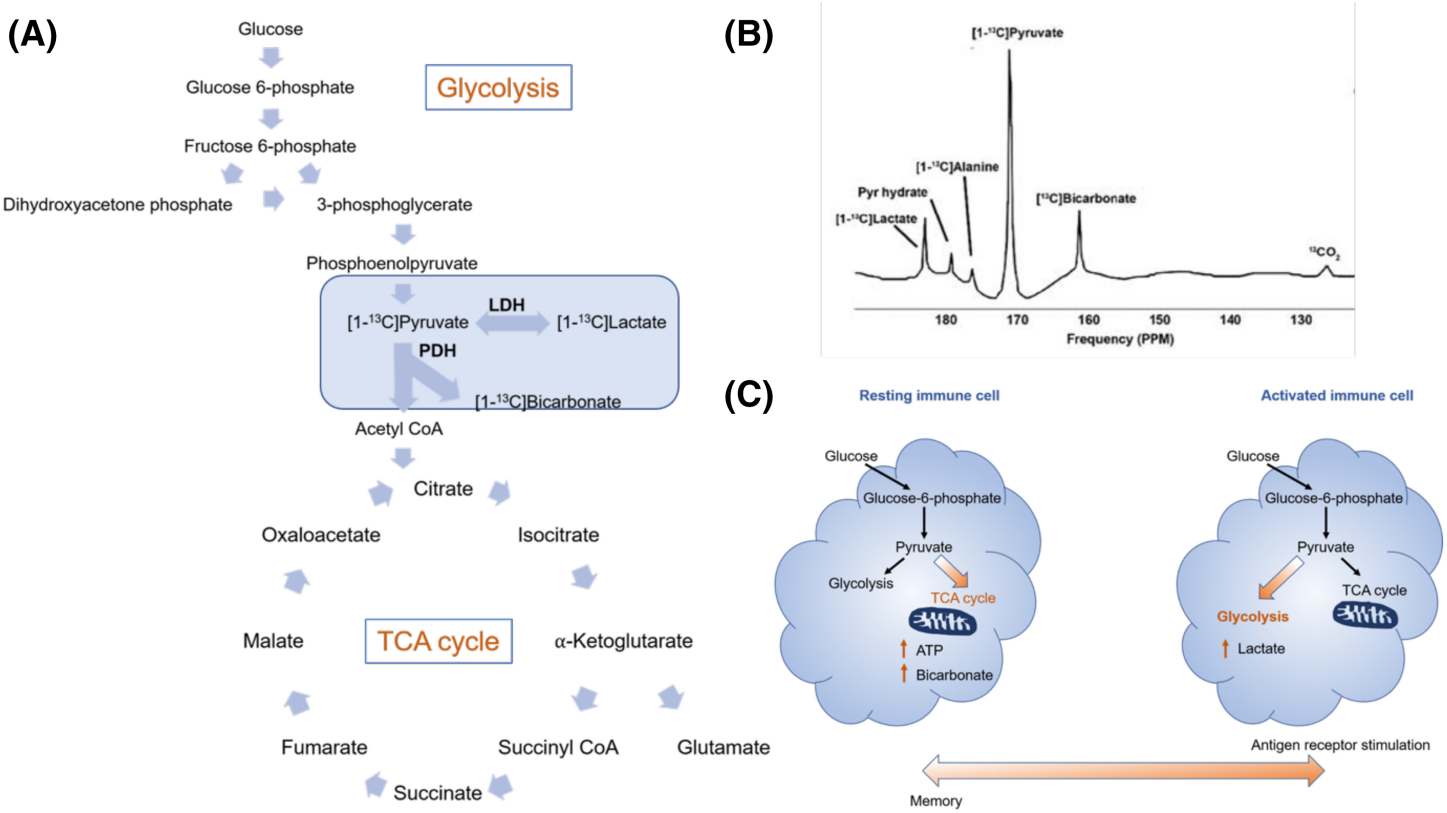
A, Metabolic flux through glycolysis and into the tricarboxylic acid cycle, highlighting flux through lactate dehydrogenase (LDH) from the interconversion of hyperpolarized [1‐^13^C]pyruvate to [1‐^13^C]lactate. B, Spectrum generated in the heart from [1‐^13^C]pyruvate flux through LDH. C, Activation of immune cell in response to antigen receptor stimulation promoting the metabolic switch of pyruvate flux from the TCA to flux through glycolysis leading to increased production of lactate

Metabolic molecules that undergo hyperpolarization using the dynamic nuclear polarization (DNP) method can be used to assess intermediates of key metabolic pathways in vivo.^39^ [1‐^13^C]pyruvate is the most studied molecule for DNP hyperpolarization techniques and occupies a central position in mammalian metabolism (Figure 1A).^17^, ^40^ Pyruvate is the end product of glycolysis and is primarily converted to acetyl‐CoA and carbon dioxide in the mitochondria via pyruvate dehydrogenase (PDH), where acetyl‐CoA then enters the tricarboxylic acid (TCA) cycle. [1‐^13^C]pyruvate flux through PDH has been used as a hallmark of viability by providing rates of continued cellular respiration through the TCA cycle.^41^ Alternatively, pyruvate can be converted to lactate via lactate dehydrogenase (LDH). While glycolysis is an essential part of ATP generation, it is normally coupled to oxidative phosphorylation to maximize ATP yield per glucose molecule. Glycolysis in the absence of oxidative phosphorylation (anaerobic glycolysis) occurs in cells experiencing metabolic stress or oxygen deficiency, although it is also seen in highly proliferative cells such as tumor cells (the Warburg effect or aerobic glycolysis) and leads to an increase in the lactate pool size and higher [1‐^13^C]lactate signal on hyperpolarized MRI.^42^ A similar pattern of metabolic reprogramming is now known to occur in numerous immune cell classes following activation.^43^

In general, activation of cells from major immune cell classes by pro‐inflammatory stimuli is associated with a metabolic switch towards anaerobic glycolysis, which is essential for their inflammatory function (Figure 1).^44^, ^45^ These pro‐inflammatory stimuli include damage‐associated molecular patterns (DAMPs), which are detected by pattern recognition receptors (PRRs) on immune cells. For example, macrophages have a central role in innate immunity and markedly increase their rate of glycolysis when activated by pro‐inflammatory signals such as lipopolysaccharides (LPS).^46^ Macrophages display an inherent ability to polarize their phenotype from pro‐inflammatory, the so‐called M1 state, to the more antiinflammatory M2 state, depending on their environment.^47^ In macrophages, increased glycolysis is coupled with increased expression of pro‐inflammatory cytokines.^47^ The pro‐inflammatory M1 state is associated with increased glycolysis while the anti‐inflammatory M2 phenotype is associated with a switch towards fatty acid oxidation.^48^ Similarly, T cell populations also show dynamic metabolic plasticity associated with changing functions. T lymphocytes are also crucial regulators of both the innate and adaptive arms of immunity and rapidly increase their metabolic capacity to sustain proliferation and function when activated.^49^ Quiescent memory, naïve and regulatory T cell subsets predominately rely on fatty acid oxidation to maintain their energy demands; however, the increased energy requirements of activated T cell populations lead to a metabolic shift towards aerobic glycolysis with a concurrent increase in lactate production.^50^, ^51^ Increased glycolysis in immune cells such as T and B cell populations is a marker of proliferation.^52^ Immunometabolic phenotyping is a dynamic and evolving area of research but provides fundamental evidence that metabolic profiles can be used to infer inflammatory cell phenotypes.^44^ Importantly for hyperpolarized MRI, the distinctions in immunometabolism enable us to infer immune cell function based on the metabolic flux of key pathways such as glycolysis. [1‐^13^C]pyruvate flux through LDH as a marker of glycolysis can be used to monitor inflammatory immune cell function in vivo.

## 3 ASSESSMENT OF IMMUNOMETABOLIC RESPONSES BY HYPERPOLARIZED MR IN ISOLATED PREPARATIONS OF IMMUNE CELLS

**FIGURE 2:**
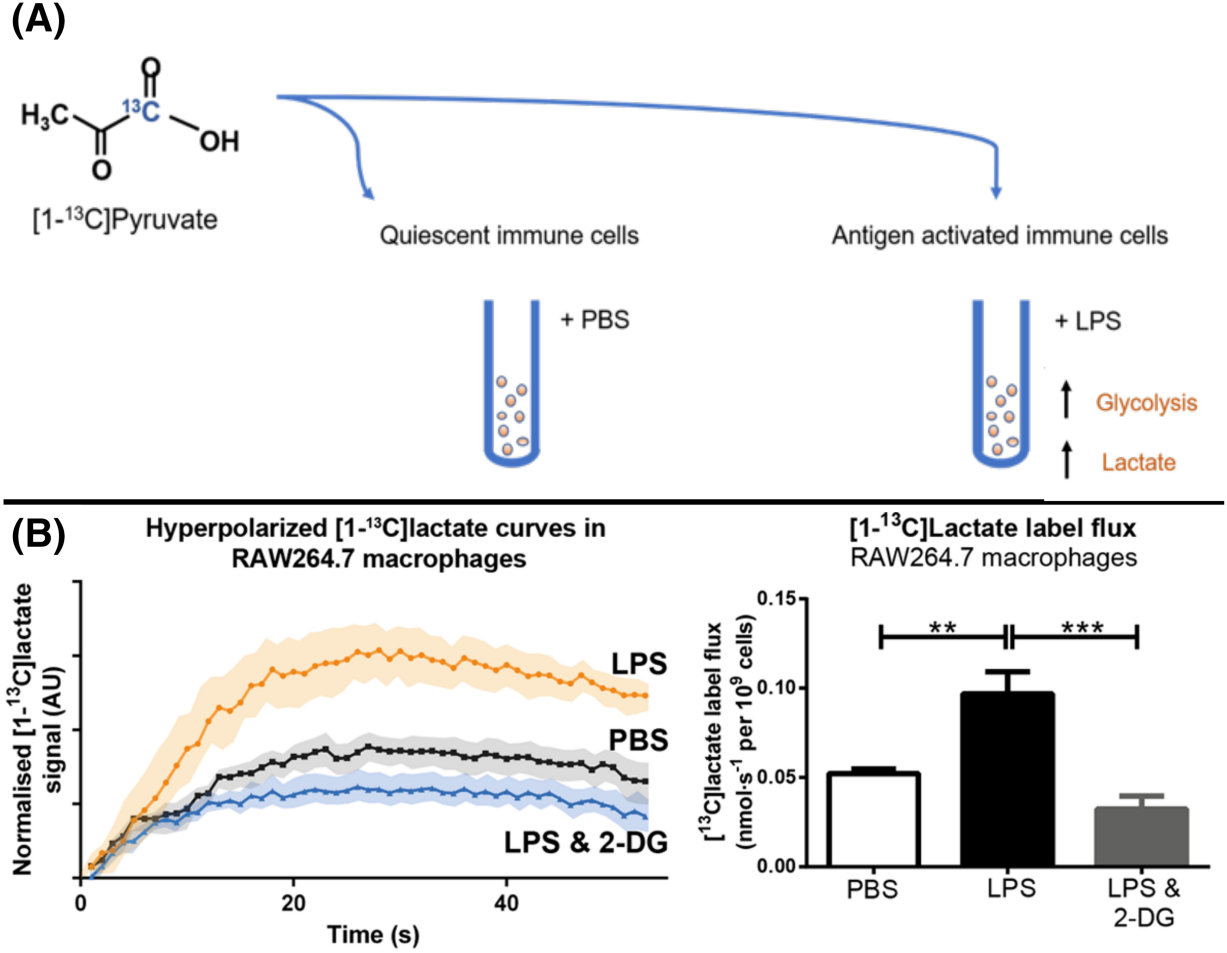
A, in vitro activation of immune cells with pro‐inflammatory stimulus lipopolysaccharide (LPS) compared with quiescent immune cells given phosphate buffered saline (PBS). B, Hyperpolarized [1‐^13^C]pyruvate flux through LDH produces higher [1‐^13^C]lactate signals in LPS‐stimulated macrophages compared with control, unstimulated macrophages^53^

Experiments using preparations of cultured immune cells have established the concept of using hyperpolarized MR to detect changes in immune cell metabolism in response to pro‐inflammatory signals and following immunomodulatory drug treatment in controlled conditions (Figure 2A). The distinct glycolytic phenotype of M1 macrophages was exploited in a study of inflammatory behavior in a mouse cell line.^54^ Hyperpolarized [1‐^13^C]pyruvate was used to assess glycolytic flux in response to pro‐inflammatory stimulation with LPS, a classical M1 activator, in mouse macrophages. Elevated lactate levels were detected in the M1 polarized cells. The M1 pro‐inflammatory phenotype was confirmed by measurement of inflammatory markers, including nitric oxide production, a potent regulator of inflammatory functions and metabolic markers of inflammation.^55^ Proton spectroscopy of control and LPS‐stimulated macrophages showed a significant increase in glycolytic metabolites, particularly in lactate production (Figure 2).^54^ Hyperpolarized [1‐^13^C]pyruvate spectroscopy similarly demonstrated a significant elevation in the lactate/pyruvate ratio in LPS‐stimulated cells.^54^ Increased [1‐^13^C]lactate production was associated with increased expression of LDH and increased levels of the cofactor NADH.^54^ Decreased expression of the monocarboxylate transporter (MCT), which regulates transportation of lactate out of the cell, led to an increase in retained, intracellular lactate.^54^ Macrophages were also treated with indomethacin, a nonsteroidal anti‐inflammatory drug, to examine the potential application of monitoring lactate production as a marker of therapeutic response to anti‐inflammatory interventions. The hyperpolarized lactate/pyruvate ratio was significantly reduced, but the mRNA expression levels of LDH were unchanged by the treatment. This study demonstrated the promising application of hyperpolarized [1‐^13^C]lactate production as a measure of inflammation‐mediated glycolytic flux in immune cells.^54^

Similarly, [1‐^13^C]pyruvate MR spectroscopy in macrophage like cell suspensions confirmed that increased glycolytic metabolism in macrophages was responsible for the increased production of lactate under pro‐inflammatory conditions.^53^ Macrophage like cells were treated with lipopolysaccharide to produce an M1, pro‐inflammatory phenotype. M1 polarization doubled lactate label flux rates. Inhibiting glycolysis with 2‐deoxyglucose normalized lactate flux and reduced the expression of pro‐inflammatory cytokines in the macrophagelike cells.

Activation of regulatory T cells rapidly increases their metabolic demand to sustain proliferation and function; this is largely met by upregulation of glycolysis. A recent study of soluble anti‐CD28–activated human T cells demonstrated the applicability of hyperpolarized ^13^C MRS for measurements of immune cell function.^56^ Stimulated regulatory T cells exhibit a similar pattern in their metabolic shift to that seen in macrophages, with an upregulation of glycolysis leading to increased lactate production.^56^ This metabolic signature was detectable through hyperpolarized [1‐^13^C]pyruvate to [1‐^13^C]lactate flux in an in vitro T cell culture.^56^ As with the macrophage models, increased [1‐^13^C]lactate (the sum of both intracellular and extracellular lactate) production was associated with increased expression of LDH and an increased lactate/pyruvate ratio in stimulated cultures compared with resting state T cells.^54^, ^56^

In a slightly different approach, inflammatory myeloid‐derived suppressor cells (MDSCs), which are involved in tumor development, were assessed by the novel application of hyperpolarized [6–^13^C]arginine.^57^ MDSCs promote tumor growth by inhibiting the anti‐tumor actions of the innate T cell‐mediated response.^58^, ^59^ Arginine is essential for T‐cell proliferation and can be broken down into urea and ornithine by arginase.^58^ High expression of arginase by MDSCs and targeted breakdown of arginine is how these inflammatory cells interfere with the T cell‐mediated response to tumor development.^60^ Using hyperpolarized [6–^13^C]arginine it is possible to measure the production of hyperpolarized [6–^13^C]urea and correlate it to arginase concentrations and hence the activity of MDSCs in vitro.^57^ In comparison with control bone marrow cells, hyperpolarized [^13^C]urea production was significantly higher in MDSCs, demonstrating that hyperpolarized [6–^13^C]arginine has potential application in the imaging of inflammatory cells in the tumor environment.^57^

These experiments collectively establish the concept of using hyperpolarized MRI to detect changes in immune cell metabolism resulting from activation with pro‐inflammatory stimuli, as well as changes in response to experimental therapy. Corroborative findings have also now been demonstrated in more complex preclinical animal models of inflammation.

## 4 HYPERPOLARIZED MRI IN ANIMAL MODELS OF INFLAMMATION AND INFLAMMATORY DISEASES

### 4.1 Inflammation in the cardiovascular system

Myocardial infarction (MI) results in the loss of large numbers of cardiomyocytes and remains a leading cause of heart failure and death worldwide.^61^ MI leads to an intense local inflammatory response in the injured myocardium, characterized by high numbers of monocytes and macrophages.^62^ Some local inflammatory response following myocardial infarction is essential for wound healing and scar formation although an excessive inflammatory response probably predisposes to excessive tissue injury and adverse cardiac remodeling and may be a therapeutic target.^63^

**FIGURE 3:**
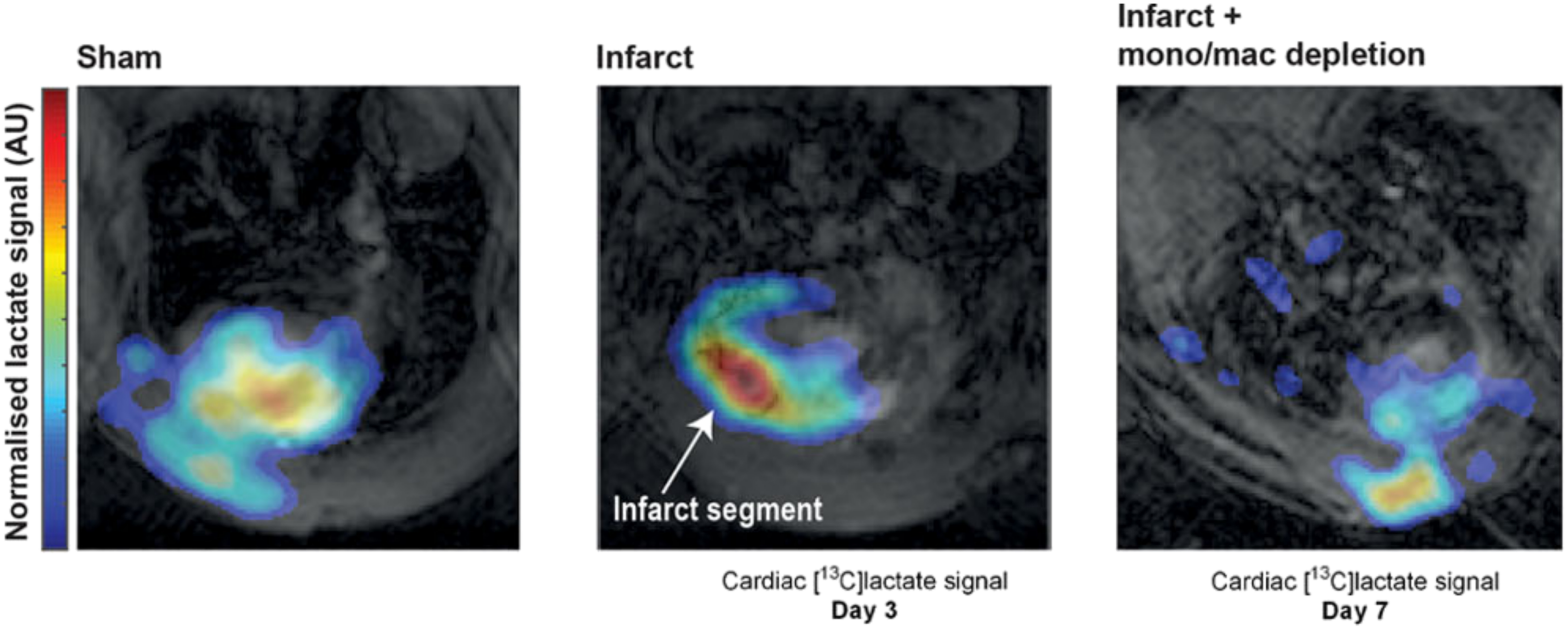
Hyperpolarized [1‐^13^C]lactate generation in a rodent model of cryoinfarction at 3 and 7 days postexperimental myocardial infarction^53^

Hyperpolarized MR using [1‐^13^C]pyruvate was applied in a preclinical study assessing inflammation following experimentally induced MI in rats by examining the spatially averaged [1‐^13^C]lactate signal.^53^ At day 3 and day 7 following MI, there was large [1‐^13^C]lactate signal in injured myocardial segments. The increase in [1‐^13^C]lactate signal intensity was abrogated when macrophage/monocyte populations were depleted from the myocardium pharmacologically, implying that immune cells (rather than the injured cardiomyocytes themselves) were the primary determinant of the [1‐^13^C]lactate signal (Figure 3). Systemic administration of the glycolytic inhibitor 2‐deoxyglucose, which is known to have anti‐inflammatory activity in cultured macrophages,^64^ also reduced the [1‐^13^C]lactate signal arising from immune cells in the injured myocardium and was associated with a reduction in pro‐inflammatory cytokines and improved cardiac remodeling.^53^ These experiments establish the feasibility of using hyperpolarized MR to assess inflammation in the cardiovascular system and provide proof of concept for “MR visible” immunomodulation using drugs. Whether hyperpolarized MR using [1‐^13^C]pyruvate can also assess immune cell activity in other cardiovascular diseases with important inflammatory components such as atherosclerosis and myocarditis remains to be tested.^65^, ^66^

Hyperpolarized MR can also be used to probe regional necrosis as a method of assessing the active phases of the inflammatory response to tissue damage. The novel tracer [1,4‐^13^C]fumarate was used to image cardiac necrosis following myocardial infarction.^67^ Cardiomyocyte necrosis was detectable 1 day after MI, with a measurable 82‐fold increase in [1,4‐^13^C]malate production that remained elevated 1 week after MI. Necrosis is closely coupled to the acute inflammatory response.^68^ By exploiting this fundamental, physiological relationship between necrosis and inflammation, this application of hyperpolarized MRI has the potential to enhance our understanding of the inflammatory response to tissue damage following MI in the acute period when interventional therapies are likely to be the most efficacious.

### 4.2 Inflammation in the CNS

A number of baseline studies have already characterized the application of hyperpolarized [1‐^13^C]pyruvate and [2‐^13^C]pyruvate MR in the study of brain metabolism.^69^, ^70^, ^71^ Imaging of [1‐^13^C]pyruvate cerebral metabolism has shown the exchange to lactate and metabolism to bicarbonate within the CNS.^71^ The brain retains its own distinct population of macrophages, which are known as microglia. This tissue‐resident population, albeit separated from other immune cell populations by the blood‐brain barrier, still functions in a remarkably similar way to general macrophage populations, especially in terms of their dynamic metabolic phenotype. Increased lactate production from microglial populations is associated with increased glycolytic activity and can be measured as an indicator of inflammatory functions within the brain.

Beyond the healthy brain, neurological pathologies such as traumatic brain injury (TBI), MS, neuro myelitis optica (NMO), depression and stroke are known to have significant immune cell involvement and altered metabolic profiles on ^1^H MRS.^72^, ^73^, ^74^, ^75^, ^76^ In TBI, neurodegenerative diseases and stroke, the innate immune system of the brain (microglia and astrocytes) respond with rapid proliferation and polarization towards both pro‐inflammatory and anti‐inflammatory states.^72^ This in turn leads to elevated glycolytic metabolism and an upregulation of lactate production to fuel rapid cellular division. The picture of the innate immune proliferative response to injury within the CNS is complex, and highly time‐dependent from the initial pathological event. For example, acute activation and proliferation of microglia in TBI initially perform an anti‐inflammatory role within the CNS to restore homeostasis; however, chronic activation can lead to further cerebral tissue damage and neurodegeneration.^72^ Conditions involving the adaptive immune system, such as MS and NMO, involve the rapid upregulation of glycolytic metabolism to fuel proliferation in response to proteins such as myelin basic protein (MBP).^77^, ^78^ PET tracers commonly used to study the innate immune system need to focus on the use of markers such as translocator protein (TSPO), which binds to an outer mitochondrial membrane protein with low affinity in quiescent tissues^79^ rather than ^18^F[FDG], due to the high background glucose metabolism of the brain.^80^

Noninvasive imaging techniques are essential for clinical neurological studies. Hyperpolarized [1‐^13^C]pyruvate studies have already shown excellent promise for detailed in vivo imaging of brain metabolism that can readily be applied to suitable clinical environments.^71^, ^81^, ^82^ Several studies have demonstrated the feasibility of using hyperpolarized [1‐^13^C]pyruvate to study glycolytic flux in the brain across different models associated with neuroinflammation. For example, hyperpolarized [1‐^13^C]lactate imaging in models of TBI revealed increased lactate production in acute and chronic periods following traumatic injury. TBI is associated with increased susceptibility to chronic neuroinflammatory and degenerative changes with deleterious outcomes including cognitive decline, loss of motor function, reduced quality of life and increased mortality.^83^ Prolonged inflammatory activity following TBI is associated with increased microglial cell number and phagocytotic activity.^84^ While the lactate/pyruvate ratio was increased, the lactate/bicarbonate ratio was reduced, implicating an increase in glycolysis, with a concomitant decrease in oxidative phosphorylation.^85^ Further studies examining the depletion of microglia populations revealed that the increased hyperpolarized [1‐^13^C]lactate signal was largely contributed to by these populations, particularly at acute time points.^86^ Importantly, it was chronic activation of microglia that was associated with cognitive decline and memory deficits in mouse models of TBI.^84^ Focal contusion models of TBI further demonstrate that the upregulation of inflammatory microglia is associated with prolonged breakdown of the blood brain‐barrier, corresponding to memory deficits at 30 days following injury. Increased glycolytic flux, associated with microglia activation, is also linked to cognitive decline in diabetic models. A mouse model investigating cognitive impairment and metabolic dysfunction associated with diabetes applied hyperpolarized [1‐^13^C]pyruvate to monitor lactate flux and found that significantly increased production of [1‐^13^C]lactate in the hippocampal region of the brain was associated with cognitive decline.^87^

Similarly, pro‐inflammatory mononuclear phagocytes were shown to contribute to neuroinflammatory lesions in a cuprizone‐mediated model of demyelination.^88^ Pro‐inflammatory microglia were also responsible for an increased hyperpolarized lactate/pyruvate ratio. In demyelinating regions there was concurrent detection of high [1‐^13^C]lactate levels and high population numbers of microglia. These macrophages were found to upregulate their expression of pyruvate dehydrogenase kinase 1 (PDK1), resulting in an inhibition of PDH with increased flux through LDH, mediating lactate production. Transgenic mice lacking immune receptor CX3 chemokine receptor 1 (CX3CR1) were unable to reproduce this response due to their deficiency in immune response, highlighting the application of hyperpolarized [1‐^13^C]pyruvate in monitoring the inflammatory response in vivo.^88^

**FIGURE 4:**
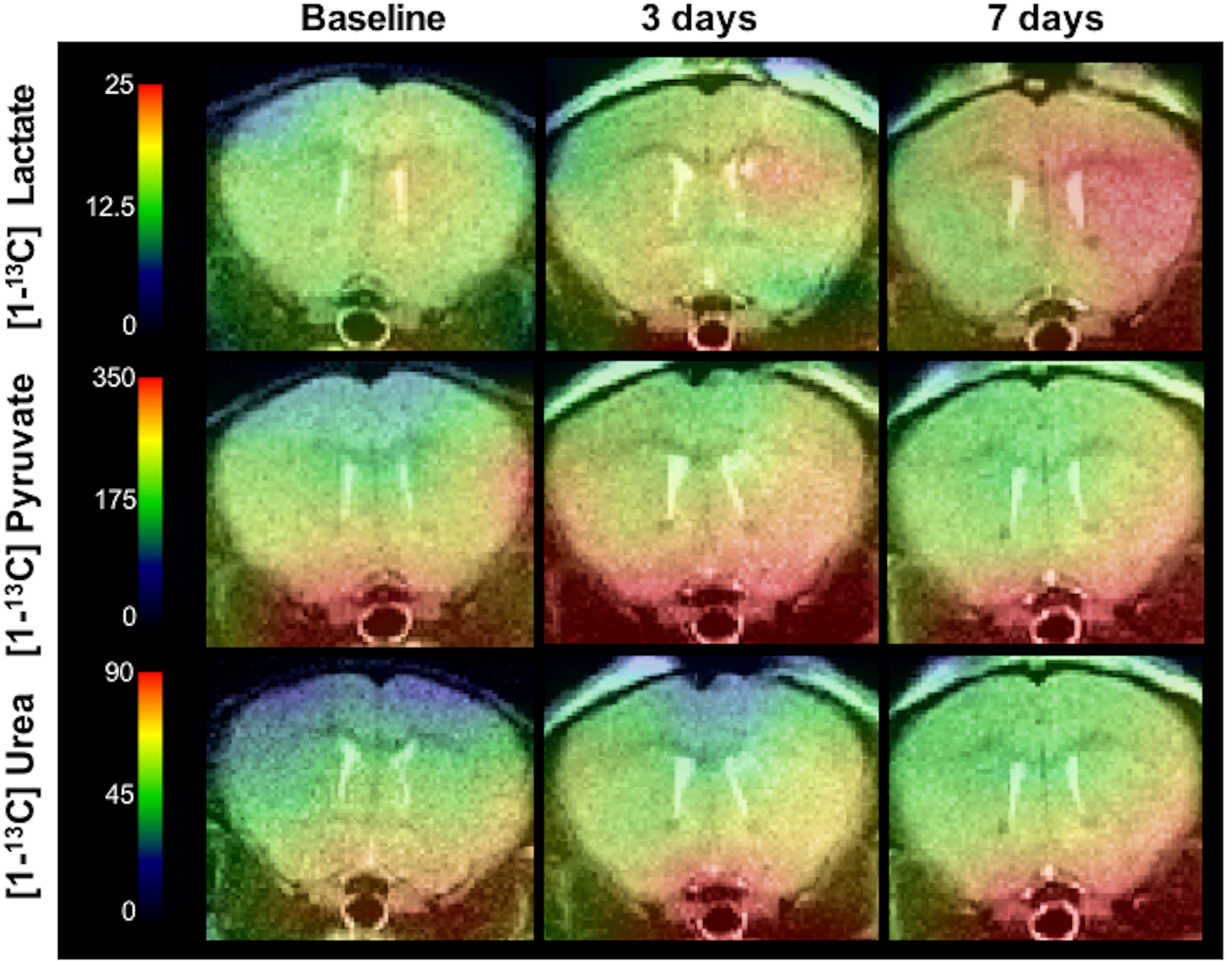
Heatmaps of hyperpolarized [1‐^13^C]lactate, [1‐^13^C]pyruvate and [1‐^13^C]urea in rodent model of neuroinflammation induced by intracranial injection of lipopolysaccharide^89^

Finally, stimulation of the pro‐inflammatory phenotype using LPS injection was applied to a mouse model of intracranial inflammation to examine the metabolic changes associated with directly induced neuroinflammation (Figure 4). Increased hyperpolarized [1‐^13^C]pyruvate flux through LDH produced a significantly higher lactate/pyruvate ratio and was associated with significantly increased microglial cell numbers.^89^ Collectively, these studies highlight a growing body of evidence that upregulated glycolysis, as detected by increased [1‐^13^C]lactate production, is a potent indicator of increased macrophage function in pathological states, and that hyperpolarized [1‐^13^C]pyruvate can be applied to measure neuroinflammation. However, it has yet to be shown that a model demonstrating T and B cell‐driven pathology, as is found in MS, has the same metabolic impairment.

### 4.3 Inflammation in the musculoskeletal system

A rodent model of inflammatory arthritis demonstrated the applicability of [1‐^13^C]pyruvate flux though LDH to measure arthritis in inflamed hind paws.^90^ Conversion of [1‐^13^C]pyruvate to [1‐^13^C]lactate was increased in arthritic paws compared with control paws, indicating a potential clinical application of hyperpolarized MRI in the diagnosis and monitoring of inflammatory arthritis. A similar study was performed in an experimentally induced murine model of rheumatoid arthritis, where [1‐^13^C]lactate signal was shown to precede inflammatory immune cell recruitment to the arthritic limb.^91^ The gradual recruitment of phagocytic immune cells was correlated to increased swelling of the affected joints while the lactate signal was highest at the earliest time points of immune cell recruitment.

### 4.4 Inflammation in the lungs

Hyperpolarized [1‐^13^C]lactate imaging has also been applied to the in vivo assessment of pulmonary inflammation.^9^, ^92^ In a bleomycin‐induced mouse model of lung inflammation, LDH activity was significantly upregulated with the [1‐^13^C]lactate signal increase accompanied by an increased presence of neutrophils in pulmonary tissues.^9^ The increased [1‐^13^C]lactate signal demonstrated an observable increase in uptake and metabolism of [1‐^13^C]pyruvate largely mediated by the increased metabolic demand of the infiltrating and expanding neutrophil population. Further characterization of the enzymatic kinetics of the LDH‐catalyzed interconversion of pyruvate to lactate revealed that the rate constant could be used to differentiate between control and inflamed lungs.^92^ A positive linear correlation between [1‐^13^C]lactate signals and neutrophil count demonstrated an ~3‐fold increase in average lactate in the inflamed lung group compared with control, further supporting that increased pro‐inflammatory immune cell behavior can be inferred from increased [1‐^13^C]lactate MR signals.

### 4.5 Inflammation in the kidneys

Hyperpolarized [1‐^13^C]pyruvate was also used to noninvasively measure metabolic markers in the kidneys of a mouse model of partial unilateral ureteral obstruction (pUUO).^10^ Surgically induced partial obstruction of the left ureter resulted in increased inflammatory immune cell recruitment to the obstructed kidney, but not to the unobstructed control kidney. Increased macrophage infiltration was associated with injury and inflammation in the obstructed kidney, and while both kidneys showed an increase in [1‐^13^C]lactate production, the obstructed kidney showed a significantly higher and sustained lactate signal, again supporting the premise that increased [1‐^13^C]lactate production can be used to infer increased immune cell inflammatory activity.

## 5 TRANSLATIONAL OUTLOOK

Inflammation is involved in the development and maintenance of numerous diseases. Studies of inflammation, particularly in cardiovascular and neurological diseases, have relied heavily on preclinical rodent models to examine the mechanisms of inflammation. While these have been invaluable, there are inherent complexities in extrapolating these to human disease states. The clinical research of inflammation has been hampered by the limitations of noninvasive tools to examine inflammatory processes. Current techniques, including ^18^F[FDG]‐PET, have been applied to image inflammation through increased glycolytic metabolism indicated by increased ^18^F[FDG] uptake; however, such techniques present limitations in terms of spatial resolution of activity and the specificity of cell types involved and are known for having additional complications involving false‐positive results.^93^ ^18^F[FDG] is also limited in that it is a good marker of tissue perfusion and uptake via glucose transporters and phosphorylation by hexokinase, but it is unable to assess downstream metabolism that can be investigated with hyperpolarized [1‐^13^C]pyruvate. To our knowledge, no human studies have yet reported the use of hyperpolarized MR with the intention of specifically imaging inflammation,^94^ although such studies are likely to follow in the near future.

As a clinically translatable technique, hyperpolarized MRI has already made tremendous progress. The first “in‐man” study using hyperpolarized MRI explored the safety and feasibility of hyperpolarized [1‐^13^C]pyruvate to assess tumor metabolism in prostate cancer patients.^95^ Subsequent follow‐up studies have also demonstrated the applicability of hyperpolarized [1‐^13^C]pyruvate spectroscopy for monitoring therapeutic responses to treatment of prostate cancer.^96^ Clinical cardiovascular applications of hyperpolarized MRI have also been demonstrated by [1‐^13^C]pyruvate flux measurements in healthy individuals.^97^, ^98^ A recent clinical trial of hyperpolarized ^13^C MR was the first to demonstrate the ability of hyperpolarized [1‐^13^C]pyruvate to assess physiological and pathological changes in metabolism in the human heart in diabetes. This is a significant first step in the translation of hyperpolarized ^13^C MRI for noninvasive assessment of cardiovascular disease. Hyperpolarized [1‐^13^C]pyruvate has also been used to quantify metabolism in the healthy human brain, revealing significantly higher metabolic rates in gray matter compared with white matter and highlighting the applicability of this technique for potential assessment of pathological cerebral metabolism.^71^ Recent application of hyperpolarized [1‐^13^C]pyruvate MRI in the field of oncology demonstrated the feasibility of measuring molecular characteristics of breast tissue tumors through [1‐^13^C]pyruvate metabolism.^99^ Increased [1‐^13^C]lactate signal was associated with more aggressive tumors through increased expression of MCT‐1. This study also highlighted the very low production of [1‐^13^C]lactate signal in nontumorigenic, normal tissue, supporting the capacity of hyperpolarized [1‐^13^C]pyruvate MRI for measuring specific areas of metabolic interest with high spatial resolution.^99^ The success of these studies in demonstrating the capability of hyperpolarized [1‐^13^C]pyruvate to measure metabolic changes in clinical studies across various physiological and pathophysiological domains supports its application to also measuring inflammation‐mediated metabolic changes in humans.

There are, of course, limitations to hyperpolarized [1‐^13^C]pyruvate MRI, notably the specificity of the [1‐^13^C]lactate signal produced. Lactate is observable in almost all normal tissues, and, as such, distinguishing lactate generated from activated immune cells from that generated by normal baseline tissue function is inherently difficult. However, with inflamed tissue there is an increased activation of resident immune cells and a significant increase in population numbers due to infiltrating immune cells that contribute to generation of higher [1‐^13^C]lactate signal intensity; this noticeable difference in focal [1‐^13^C]lactate generation helps to distinguish the magnitude of the contribution of immune cell types to changes in metabolic activity in areas of interest compared with normal tissue.^53^

Further confirmation of the contribution made to [1‐^13^C]lactate signal from immune cells can be determined by depleting those immune cells populations and re‐examining the [1‐^13^C]lactate signal. For example, monocyte/macrophage populations can be depleted using clodronate liposomes; application of this in a rat MI model demonstrated the significant contribution of these cells to the [1‐^13^C]lactate signal intensity generated in the infarcted region of the myocardium.^53^

Other hyperpolarized MR agents with potential applications for imaging inflammation include using hyperpolarized glucose or hyperpolarized choline probes. Hyperpolarized [U‐^2^H, U‐^13^C]glucose studies have been developed for applications in oncology for monitoring evidence of tumor treatment response and tumor pentose phosphate pathway.^100^ This approach could potentially be applied to monitoring glycolytic flux in immune cells, with possible translatability to human patients. Deuterated hyperpolarized choline chloride is also a promising DNP probe with comparable biophysical properties to pyruvate.^31^ The nonradioactive 2DG analogue, [^13^C_6_, D_8_]2DG, is another potential alternative for noninvasive monitoring of glycolytic flux.^101^

Future studies will compare the performance of hyperpolarized MR for inflammation imaging with existing techniques such as ^18^F[FDG]‐PET, although hyperpolarized MR has potential advantages, including more rapid acquisition times and an absence of ionizing radiation, which could enable longitudinal studies and therapy tracking in the same patient. Balanced against this, clinical hyperpolarization equipment is much less widely available than PET and whether the maximum spatial resolution of hyperpolarized MR will match that of PET is currently unclear. In addition, PET tracers with a greater degree of immune cell specificity are available (including PBR2836, PK1119537 and ^68^Ga‐DOTATATE, among others; eg, for macrophage imaging), and the optimal technique is likely to depend upon the specific clinical or research question to be answered.

## 6 CONCLUSIONS

Hyperpolarized ^13^C MRI is a novel, clinically applicable method that can be used to measure physiological and pathological metabolic fluxes in real time in vivo. Preclinical studies have outlined the potential applications of this technique for assessing inflammation by exploiting characteristic metabolic shifts in activated immune cells, primarily with the use of [1‐^13^C]pyruvate to detect increased label incorporation into [1‐^13^C]lactate. With the clinical translatability of hyperpolarized MRI already outlined in the successful clinical studies thus far achieved, future clinical applications of this technique across different pathological domains appear highly attainable. Inflammatory physiology is undeniably important across many disease states yet inherently difficult to study in humans. By using hyperpolarized [1‐^13^C]pyruvate to study glycolytic metabolism associated with increased inflammatory cell activity it may be possible to enhance our understanding of inflammatory‐driven disease processes. Future studies focused on the application of hyperpolarized MR with [1‐^13^C]pyruvate may provide important insights into the inflammatory processes of cardiac and neurological diseases and offer new, noninvasive clinical methods for monitoring and improving our understanding of chronic inflammatory diseases.

## FUNDING INFORMATION

British Heart Foundation FS/19/18/34252 RE/18/3/34214European Commission 858149 National Institute for Health Research Oxford Biomedical Research Centre.
